# Trp31 Residue of Trx-1 Is Essential for Maintaining Antioxidant Activity and Cellular Redox Defense Against Oxidative Stress

**DOI:** 10.3390/antiox14030257

**Published:** 2025-02-24

**Authors:** Zongmao He, Yi Yan, Xijun Guo, Tong Wang, Xinqiao Liu, Ren-Bo Ding, Yuanfeng Fu, Jiaolin Bao, Xingzhu Qi

**Affiliations:** 1 Key Laboratory of Tropical Biological Resources of Ministry of Education, School of Pharmaceutical Sciences, Collaborative Innovation Center of One Health, Hainan University, Haikou 570228, China; 22211007000004@hainanu.edu.cn (Z.H.); 22211007000025@hainanu.edu.cn (Y.Y.); guoxu@hainanu.edu.cn (X.G.); b2024010032@student.pumc.edu.cn (T.W.); liuxq225@mail2.sysu.edu.cn (X.L.); dingrenbo@hainanu.edu.cn (R.-B.D.); 23211007000048@hainanu.edu.cn (Y.F.); 2 School of Pharmaceutical Sciences, Sun Yat-sen University, Guangzhou 510006, China; 3 State Key Laboratory of Quality Research in Chinese Medicine, Institute of Chinese Medical Sciences, University of Macau, Macao 999078, China

**Keywords:** thioredoxin-1, Trp31 residue, antioxidant activity, redox defense, disulfide bond

## Abstract

Thioredoxin-1 (Trx-1) is an important redox protein found in almost all prokaryotic and eukaryotic cells, which has a highly conserved active site sequence: Trp-Cys-Gly-Pro-Cys. To investigate whether the Trp31 residue is essential for the antioxidant activity of human Trx-1 (hTrx-1), we mutated Trx-1 by replacing Trp31 with Ala31 (31Ala) or deleting Trp31 residue (31Del). We introduced 31Ala and 31Del mutations into prokaryotic cells for hTrx-1 protein expression, protein purification and evaluation of antioxidant activity. The results showed that neither the replacing mutation to Ala31 nor the deletion of Trp31 residue affected the efficient expression of hTrx-1 protein in prokaryotic cells, indicating that neither form of Trp31 mutation would disrupt the folded structure of the Trx-1 protein. Comparison of the antioxidant activity of purified hTrx-1 proteins of wild-type, 31Ala and 31Del forms revealed that both mutant forms significantly decreased the antioxidant capacity of hTrx-1. Further investigations on eukaryotic cells showed that H_2_O_2_ treatment caused massive cell death in EA.Hy926 human endothelial cells with 31Ala and 31Del mutations compared to wild-type cells, which was associated with increased ROS production and downregulation of antioxidant Nrf2 and HO-1 expression in the mutant cells. These results suggested that mutations in the Trp31 residue of hTrx-1 remarkably disrupted cellular redox defense against oxidative stress. The antioxidant activity of hTrx-1 relies on the thiol–disulfide exchange reaction, in which the content of thiol groups forming disulfide bonds in hTrx-1 is critical. We found that the content of free thiol groups specifically participating in disulfide bond formation was significantly lower in Trp31 mutant hTrx-1 than in wild-type hTrx-1; that was speculated to affect the formation of disulfide bonds between Cys32 and Cys35 by virtual analysis, thus abolishing the antioxidant activity of hTrx-1 in cleaving oxidized groups and defending against oxidative stress. The present study provided valuable insights towards understanding the importance of Trp31 residue of hTrx-1 in maintaining the correct conformation of the Trx fold structure, the antioxidant functionality of hTrx-1 and the cellular redox defense capability against oxidative stress.

## 1. Introduction

Thioredoxin-1 (Trx-1) is a low molecular weight (12 kDa) redox protein, which belongs to the thioredoxin family and presents in nearly all prokaryotic and eukaryotic cells. Trx-1 protein features a highly conserved active site sequence: Trp-Cys-Gly-Pro-Cys [1]. Trx-1, together with thioredoxin reductase (TrxR) and NADPH, forms the thioredoxin system. In this system, NADPH serves as the electron donor, while Trx and TrxR represent two different types of antioxidant oxidoreductase proteins [2]. The thioredoxin system is an effective reductant of disulfide bonds in target proteins, through the thiol–disulfide exchange reaction. During the process, the cysteine residues at positions 32 and 35 in Trx-1 protein are oxidized and form an intramolecular disulfide bond [3]. Therefore, the content of thiol groups forming disulfide bonds in hTrx-1 is critical to its antioxidant activity.

Previous studies have determined the 3D structure of Trx-1 proteins in multiple species, which have a very similar and stable 3D structure known as the Trx fold. Its center is a hydrophobic core, composed of five β-sheets, surrounded by four α-helices outside [4,5,6,7]. The composition order of the Trx fold is β1-α1-β2-α2-β3-α3-β4-β5-α4 [8]. The conserved amino acid residues play an important role in maintaining the 3D conformational architecture and functionality of Trx-1 proteins. The active site of Trx-1 consists of a conserved amino acid sequence: Trp-Cys-Gly-Pro-Cys, located on a short protruding loop between the amino-terminal region of the β2-sheet and the α2-helix [9].

Tryptophan (Trp) is an essential amino acid playing a crucial role in protein biosynthesis [10]. As the amino acid with the highest energy cost in biosynthesis, tryptophan residues are usually considered as being buried in the core of proteins to maintain a stable 3D structure. All prokaryotic and eukaryotic thioredoxins harbor a conserved tryptophan residue, which is situated at the disulfide/dithiol active site [11]. However, there is an increasing recognition of the contribution of tryptophan sites to protein function, with Trp playing roles in processes such as protein folding and signal transduction [12,13]. For example, the irreversible oxidation of tryptophan by reactive oxygen species (ROS) can promote subsequent metal binding and/or tryptophan dimerization processes, leading to an increase or loss of protein function [14,15,16]. Meanwhile, evolutionary biologists have found that Trp is the most conserved amino acid, while other amino acids are more prone to mutations and substitutions during evolution [17]. Throughout the molecular evolution of the Trx-1, subtle changes in the Trx fold structure, particularly at the conserved active site, can lead to partial alteration in protein functionality [18,19,20].

In this study, we specifically focused on the Trp31 residue in the conserved sequence domain WCGPC of hTrx-1, to investigate its role in maintaining the correct conformation of the Trx fold structure, the antioxidant functionality of hTrx-1 and the cellular redox defense capability against oxidative stress. We mutated Trx-1 by replacing Trp31 with Ala31 (31Ala) or deleting Trp31 residue (31Del) and introduced these mutations into prokaryotic and eukaryotic cells for functional investigations. Our study would provide valuable insights towards understanding the functional importance of Trp31 residue of hTrx-1.

## 2. Materials and Methods

### 2.1. Vector Construction for Introducing Trp31 Deletion (31Del) and Substitution Mutation (31Ala) in hTrx-1

The protein sequence of human thioredoxin 1 (hTrx-1) was downloaded from the NCBI website (Accession number: AAF86466). The mutations were introduced by replacing Trp31 with Ala31 (31Ala) or deleting Trp31 residue (31Del) through editing the cDNA gene coding sequence. The hTrx-1 cDNA sequences of wild type, 31Ala and 31Del were synthesized by the Sangon Biotech (Shanghai, China) company, then were constructed into the prokaryotic expression vector pET28a and eukaryotic expression vector pCDH.

### 2.2. Prokaryotic Expression and Purification of hTrx-1 Protein

The constructed plasmids of wild type, 31Ala and 31Del were transformed into *E. coli* strain BL21 (DE3) using the CaCl_2_ transformation method. After bacterial culture in kanamycin containing LB medium for overnight culture, IPTG was added to induce Trx-1 protein expression. Then the bacterial liquid was collected and centrifuged. The collected bacteria were suspended with bacterial protein extraction solution and lysed in an ultrasonic cell disruptor. Trx-1 protein purification was conducted with the large-scale purification method for His-tagged proteins using the His-tag protein purification kit from Beyotime (Shanghai, China) company. The obtained proteins are named as follows: wild-type hTrx-1 (WT), hTrx-1-Trp31→Ala31 (31Ala) and hTrx-1-Trp31-deletion (31Del).

### 2.3. Antioxidant Activity Detection

The antioxidant activities of wild-type hTrx-1, hTrx-1-Trp31→Ala31, and hTrx-1-Trp31-deletion were detected using the Thioredoxin Activity Assay Kit (FkTRX-02-V2) purchased from IMCO Corporation Ltd., Edmonton, AB, Canada, the Total Antioxidant Capacity (T-AOC) Assay Kit from Solarbio, and the ABTS Radical Scavenging Capacity Assay Kit from Solarbio (Beijing, China). The assayed samples were bacterial protein supernatants after ultrasonic disruption. The specific operations were carried out according to the instructions provided by the kits.

### 2.4. Sulfhydryl Content Detection

The sulfhydryl contents of wild-type hTrx-1, hTrx-1-Trp31→Ala31 and hTrx-1-Trp31-deletion were determined using the Total Sulfhydryl Content Assay Kit purchased from Solarbio. For determining the sulfhydryl content forming disulfide bonds in hTrx-1, we calculated the difference of the sulfhydryl content between oxidized samples treated with 1 mmol/L tert-Butyl hydroperoxide (TBHP) and untreated samples in a reduced state.

### 2.5. ROS Content Detection

2,7-Dichlorodihydrofluorescein diacetate (DCFH-DA) was used to assess the cellular ROS production. The EA.Hy926 cells were treated with H_2_O_2_ for 12 h, after which the supernatant was discarded, and 100 μM DCFH-DA was added into each well for further 20-min incubation. Finally, the cellular ROS contents were detected using the Guava easyCyte flow cytometer (Merck Millipore, Burlington, MA, USA).

### 2.6. Western Blot Analysis

The samples were prepared using lysis buffer (P0013J, Beyotime), followed by electrophoresis on SDS-PAGE, and transferred onto PVDF membranes (SEQ00010, Merck Millipore). The membranes were incubated overnight with the following appropriate primary antibodies: Bax (1:8000, 50599-2-IG, Proteintech, Rosemont, IL, USA), HO-1 (1:1000, AF5393, Affinity Biosciences, Cincinnati, OH, USA), Nrf2 (1:1000, AF0639, Affinity Biosciences), Trx-1 (1:8000, 14999-1-AP, Proteintech), GAPDH (1:10,000, 10494-1-AP, Proteintech). Then, the secondary antibody (1:5000, SA00001-2, Proteintech) was applied for incubation of another 1 h at room temperature. The membranes were detected using ECL (WBKLS0500, Merck Millipore).

### 2.7. Cell Lines

The human umbilical vein endothelial cell line EA.Hy926 was purchased from Procell Life Science & Technology Company Limited (Wuhan, China). EA.Hy926 cells were maintained in EA.Hy926 special media (CM-0272, Procell, Wuhan, China).

### 2.8. Statistical Analysis

GraphPad Prism (version 9.4) was adopted for all the statistical analysis. Pairwise comparisons were performed via one-way ANOVA, two-way ANOVA, Tukey’s test, and *t*-test. All data with *p* < 0.05 were considered statistically significant.

## 3. Results

### 3.1. The Structural Characteristics of hTrx-1 with Trp31 Deletion and Mutation

**Figure 1:**
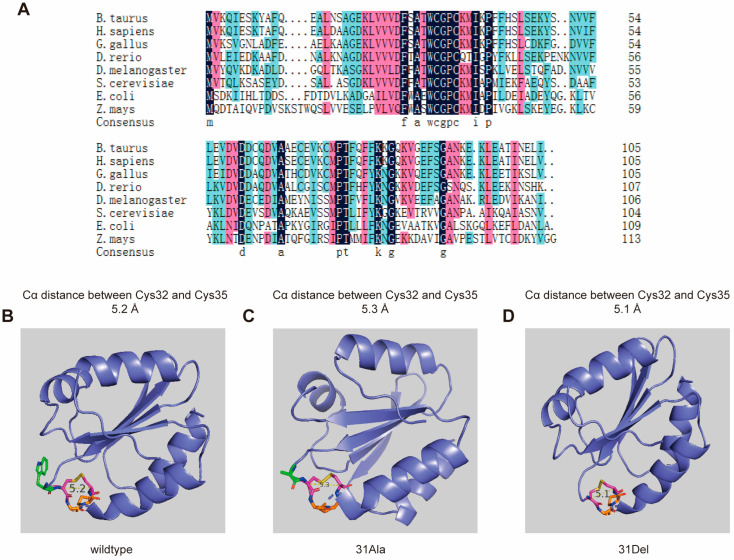
The structural characteristics of hTrx-1 with Trp31 deletion and mutation. (**A**) Sequence alignment of Trx-1 from different species. 3D structure of wild-type hTrx-1 (**B**), hTrx-1-Trp31→Ala31 (**C**), and hTrx-1-Trp31- deletion (**D**). The distances between Cα atoms of disulfide bonded cysteines at positions 32 and 35 were 5.2 Å (**B**), 5.3 Å (**C**) and 5.1 Å (**D**), respectively.

Through multiple sequence alignment of Trx-1 protein sequences from eight common species (*Bos taurus*; *Homo sapiens*; *Gallus gallus*; *Danio rerio*; *Drosophila melanogaster*; *Saccharomyces cerevisiae*; *Escherichia coli*; *Zea mays*) ranging from lower to higher organisms (Figure 1A), it was found that the redox active region of Trx-1 is highly conserved throughout the long evolution of species from bacteria to human, with the sequence Trp-Cys-Gly-Pro-Cys. Residues Cys32 and Cys35 form disulfide bonds with sulfhydryl groups on the R side chains to reduce the oxidized target protein, which is considered as being the basic function of Trx-1. Within this highly conserved motif, studies have already demonstrated the non-replaceability of Pro34 residue between Cys32 and Cys35 [21]. In this study, we investigated the Trp31 site by replacing Trp31 with Ala31 (31Ala) or deleting Trp31 residue (31Del) to explore whether it is irreplaceable in maintaining the spatial conformation and basic physiological functions of hTrx-1.

We generated the PDB files of hTrx-1 with 31Ala and 31Del mutations using the SWISS-MODEL online tool (https://swissmodel.expasy.org/, accessed on 1 October 2024), and compared their 3D structures with wild-type hTrx-1 in PyMOL software (version 2.1). The results showed that the spatial structure of the Trp31 mutant/deficient hTrx-1 was basically the same as that of the wild-type hTrx-1, with a spatial configuration of five β-folds and four α-helices. The conserved active center sequence of hTrx-1: Trp-Cys-Gly-Pro-Cys is located on a short protruding loop between the amino-terminal region of the β2-sheet and the α2-helix [9], and the Trp31 residue is located in the hydrophobic region outside the 14-membered disulfide ring composed of Cys32-Cly-Pro-Cys35 (Figure 1B). Due to the alteration in Trp31, the size of the space occupied by its R group also changes, which may affect a change in the relative positions of various functional groups near the active center site. Three-dimensional structural analysis revealed that in the reduced state, when Trp31 was deleted or mutated to Ala, the distance between the two cysteine residues Cα at positions 32 and 35 remained unchanged compared to the wild-type hTrx-1, both at 5.6 Å. However, in the oxidized state, the distance between the two cysteine residues Cα at positions 32 and 35 changed, while that of the wild type was 5.2 Å, and the distance between the two cysteine residues Cα of hTrx-1-Trp31→Ala31 became 5.3 Å, and that of hTrx-1-Trp31-deletion became 5.1 Å (Figure 1B–D). Therefore, we hypothesized that the alteration of Trp31 led to a change in the relative positions of the functional groups near the active center site, which would hinder the formation of disulfide bonds at positions 32 and 35, thereby affecting the normal antioxidant function of hTrx-1.

### 3.2. Prokaryotic Expression of hTrx-1 Protein with Trp31 Deletion and Mutation

**Figure 2:**
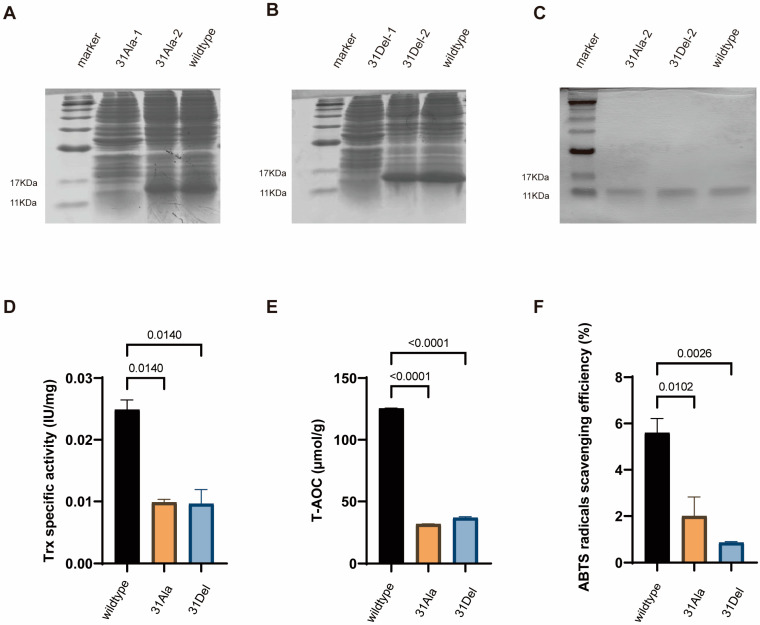
Prokaryotic expression and antioxidant activity examination of hTrx-1 protein with Trp31 deletion and mutation. SDS-PAGE electrophoresis analysis of prokaryotic protein expression products of hTrx-1-Trp31→Ala31 (**A**) and hTrx-1-Trp31-deletion (**B**). Lane 1 indicates protein marker, lane 2 indicates mutant hTrx-1 sample without IPTG induction, lane 3 indicates mutant hTrx-1 sample with 1 mmol/L IPTG induction, lane 4 indicates wildtype hTrx-1 sample with 1 mmol/L IPTG induction. (**C**) SDS-PAGE electrophoresis analysis of purified protein samples of hTrx-1-Trp31→Ala31, hTrx-1-Trp31-deletion and wild-type hTrx-1. Examination of Trx-1 activity (**D**), total antioxidant capacity (**E**) and ABTS radical scavenging ability (**F**) in wild-type hTrx-1, hTrx-1-Trp31→Ala31 and hTrx-1-Trp31-deletion proteins. n = 3. All the quantitative data are presented as mean ± SEM, and statistical significance was assessed by one-way ANOVA followed by *t* test. The *p*-value is indicated, as shown in the diagram.

We constructed the hTrx-1 gene coding sequences of wild type, 31Ala and 31Del into a prokaryotic expression vector, then transformed them into the E. coli strain BL21 for induced hTrx-1 protein expression. The hTrx-1-expressing BL21 bacteria were collected and lysed by ultrasonication, then subjected to SDS-PAGE electrophoresis on a 12% polyacrylamide gel. After staining with Coomassie Brilliant Blue, it could be observed that all target proteins were efficiently expressed, including wild-type hTrx-1, hTrx-1-Trp31→Ala31, and hTrx-1-Trp31-deletion (Figure 2A,B). All three proteins presented in the supernatant after ultrasonic disruption, indicating that they were soluble in water. Purified wild-type and mutant hTrx-1 proteins were further obtained by 0.5 and 1 mmol/L IPTG induction and extracted by a His-tag protein purification kit. The SDS-PAGE electrophoresis showed that purified prokaryotic expressed hTrx-1 proteins of wild type, 31Ala and 31Del were successfully obtained (Figure 2C).

### 3.3. Trp31 Deletion/Mutation Decreased Antioxidant Activity of hTrx-1 Protein

In the thioredoxin system, Trx-1 reduces the disulfide bonds of target proteins to thiols, helping the target proteins maintain their native structures or change conformations in response to various environmental stresses [3]. To determine whether Trp31 deletion/mutation affects Trx-1 activity, we used the classic insulin assay method, which detects the ability of hTrx-1 to reduce insulin disulfide bonds [10,12]. The results showed that both Trp31 deletion and Trp31→Ala31 alteration significantly reduced the Trx-1 activity compared with the wild type (Figure 2D). We therefore hypothesized that the deletion/mutation of Trp31 would impair the ability of Trx-1 to form intramolecular disulfide bonds at the active center site.

Furthermore, the total antioxidant capacity of wild-type and mutant hTrx-1 was measured using the FRAP method. The results indicated that both Trp31 deletion and Trp31→Ala31 alteration significantly decreased the antioxidant ability to reduce Fe^3+^-TPTZ to Fe^2+^-TPTZ compared to the wild type (Figure 2E). The ABTS radical scavenging activity were also examined, which showed that wild-type hTrx-1 exhibited significantly better radical scavenging ability than mutant hTrx-1 (Figure 2F). Our results demonstrated Trp31 deletion and Trp31→Ala31 alteration significantly impaired the antioxidant capacity of hTrx-1 protein.

### 3.4. Trp31 Deletion/Mutation Disrupted Cellular Redox Defense Against Oxidative Stress

**Figure 3:**
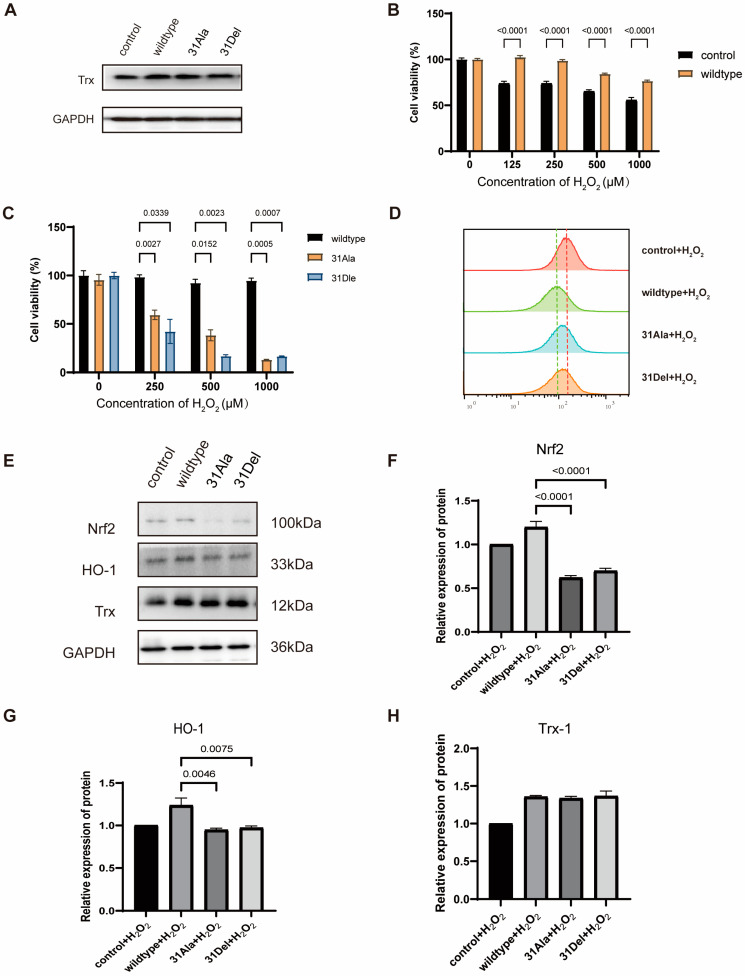
Trp31 deletion/mutation disrupted cellular redox defense against oxidative stress. (**A**) Western blot analysis of hTrx-1 expression in EA.hy926 cells transfected with empty vector (control), wild-type hTrx-1 (wildtype), hTrx-1-Trp31→Ala31 (31Ala) and hTrx-Trp31-deletion (31Del). (**B**) Comparison of cell viabilities between control and wild-type groups after treatment with 0, 125, 250, 500, 1000 μM H_2_O_2_ for 12 h (n = 3). (**C**) Comparison of cell viabilities among wild-type, 31Ala and 31Del groups after treatment with 0, 250, 500, 1000 μM H_2_O_2_ for 12 h (n = 3). (**D**) Intracellular ROS levels of control, wild-type, 31Ala and 31Del groups after treatment with 200μM H_2_O_2_ for 12 h. DCFH-DA probe was used to label intracellular ROS. The green dashed line labeling the median value of intracellular ROS level of wild-type group. The red dashed line labeling the median value of intracellular ROS level of control group. (**E**) Western blot analysis of Trx-1, Nrf2, HO-1 and GAPDH protein expression in control, wild-type and mutant EA.hy926 cells after treatment with 200μM H_2_O_2_. The densitometric analysis of Nrf2 (**F**), HO-1 (**G**) and Trx-1 (**H**) obtained from three experimental replicates. The quantitative data (**B**,**C**) are presented as mean ± SEM and statistical significance was assessed by one-way ANOVA followed by *t* test. The *p*-value is indicated, as shown in the diagram.

It has been demonstrated that accumulation of reactive oxygen species (ROS), such as H_2_O_2_, can lead to apoptosis and death of endothelial cells, and hTrx-1 was involved in defending against this oxidative stress-mediated cell death [22]. We therefore investigated whether Trp31 deletion/mutation would maintain the cellular redox defense of hTrx-1 against oxidative stress. We introduced wild-type hTrx-1, hTrx-1-Trp31→Ala31, and hTrx-1-Trp31-deletion into EA.Hy926 human endothelial cells by the lentivirus system. Compared to control cells expressing endogenous hTrx-1 and transfected with empty vector, cells overexpressed with wild-type hTrx-1, 31Ala and 31Del all exhibited elevated hTrx-1 expression with similar levels in Western blot analysis (Figure 3A). We next treated these cells with 0, 125, 250, 500 and 1000 μM of H_2_O_2_ for 12 h to explore the influences of overexpressing wild-type hTrx-1 and mutating Trp31 on cell viability under oxidative stress. The results showed that overexpression of wild-type hTrx-1 significantly improved the cell survival capability in response to oxidative stress induced by H_2_O_2_ treatment (Figure 3B). However, comparing to cells with wild-type hTrx-1, EA.Hy926 cells with Trp31 deletion/mutation exhibited significantly lower survival rates upon 0, 250, 500 and 1000 μM H_2_O_2_ treatment (Figure 3C). These results suggested that Trp31 deletion/mutation could not maintain the redox defense function of hTrx-1 against oxidative stress-mediated cell death, despite the preservation of cellular hTrx-1 protein expression.

When the level of ROS in a cell increases beyond the range of its cellular scavenging capacity, it will disrupt the cellular redox homeostasis and induce oxidative stress, ultimately leading to cell death. Therefore, we wondered whether the increased cell mortality observed in EA.hy926 cells with Trp31 deletion/mutation was associated with high intracellular ROS levels. As shown in Figure 3D, overexpression of wild-type hTrx-1 reduced intracellular ROS levels in EA.hy926 cells under 200μM H_2_O_2_ treatment, indicating that its antioxidant ability to scavenge ROS was enhanced. However, higher intracellular ROS levels were detected in EA.Hy926 cells with Trp31 deletion/mutation compared to cells with wild-type hTrx-1 upon H_2_O_2_ treatment. These results suggested that Trp31 deletion/mutation abolished the antioxidant ability of hTrx-1 to scavenge excessive ROS and disrupted cellular redox defense against oxidative stress.

To further elucidate the underlying signaling involved in Trp31 deletion/mutation-associated disruption of cellular redox defense against oxidative stress, we examined the redox signaling Nrf2-HO-1 by Western blot (Figure 3E–H). The results showed that the antioxidant proteins Nrf2 and HO-1 were both exhibited lower expression levels in H_2_O_2_-treated EA.Hy926 cells with Trp31 deletion/mutation compared to cells with wildtype hTrx-1 (Figure 3F,G). These data may explain why cells with Trp31 deletion/mutation appeared with vulnerable cellular redox defense and survival capacity under oxidative stress, which was associated with downregulation of antioxidant Nrf2-HO-1 signaling in the Trp31 mutant cells.

### 3.5. Trp31 Deletion/Mutation Inhibited the Formation of Disulfide Bonds in hTrx-1 Protein

**Figure 4:**
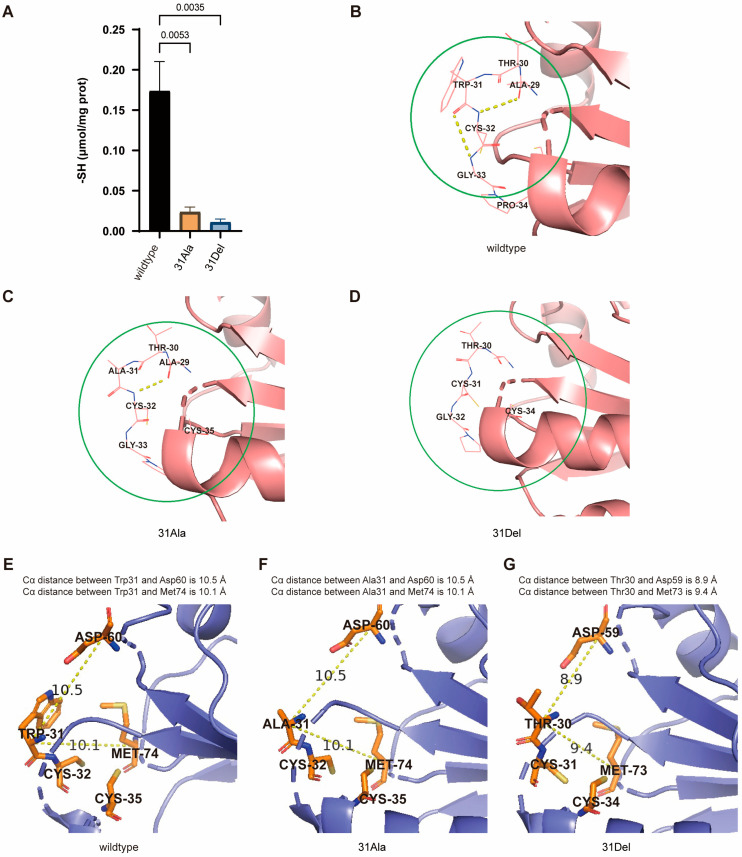
(**A**) Comparison of the thiol content involved in disulfide bond formation among the wild-type hTrx-1, hTrx-1-Trp31→Ala31 and hTrx-1-Trp31-deletion proteins (n = 3). All the quantitative data are presented as mean ± SEM, and statistical significance was assessed by one-way ANOVA followed by *t* test. The *p*-value is indicated, as shown in the diagram. (**B**–**D**) The hydrogen bond distribution of antioxidant active centers of three types of hTrx-1 in the reduced state analyzed by SPDBV software (version 4.10). In wild-type hTrx-1 (**B**), hydrogen bonds existed between the C=O of Trp31 and the N-H of Gly33, as well as between the C=O of Ala29 and the N-H of Cys32, as indicated by the yellow dashed lines; In hTrx-Trp31→Ala31 (**C**), the hydrogen bonds between the C=O of Ala29 and the N-H of Cys32 still existed, but the hydrogen bond between the C=O of Ala31 and the N-H of Gly33 disappeared, as indicated by the yellow dashed line. In hTrx-1-Trp31-deletion (**D**), the hydrogen bonds in both positions were completely absent. (**E**–**G**) The relative position changes of amino acid residues Trp31, Asp60 and Met74, near the active center site of three types of hTrx-1 under the reduced state, analyzed by Pymol software. In wild-type hTrx-1 (**E**), the Cα distance between Trp31 and Asp60 is 10.5 Å, and the Cα distance between Trp31 and Met74 is 10.1 Å. In hTrx-Trp31→Ala31 (**F**), the distance between the Cα of Ala31 and the Cα of Asp60/Met74 did not change compared to wild-type hTrx-1. In hTrx-1-Trp31-deletion (**G**), the absence of Trp31 resulted in 8.9 Å and 9.4Å distances between the Cα of Thr30 and the Cα of Asp59/Met73, respectively.

The thioredoxin system is an effective reductant of disulfide bonds in target proteins, through the thiol–disulfide exchange reaction. Thus, the formation of disulfide bonds is critical for the antioxidant activity of hTrx-1 protein. The amino acid sequence of the mammalian Trx-1 contains a total of five cysteine residues: Cys32, Cys35, Cys62, Cys69 and Cys73. Of these, Cys32 and Cys35 are exposed to the hydrophobic region of the conserved active center sequence: Trp-Cys-Gly-Pro-Cys, which is involved in the redox regulation of hTrx-1 protein, and these two cysteine residues provide the thiol groups required for thioredoxin-dependent reduction activity. To investigate whether deletion/mutation of Trp31 affected the formation of disulfide bonds in hTrx-1 protein, we detected the total free thiol content in oxidized hTrx-1 protein samples treated with tert-Butyl hydroperoxide (TBHP) and untreated samples in a reduced state. The difference in the sulfhydryl content between the oxidized and reduced-state hTrx-1 protein samples represents the thiol content forming disulfide bonds in hTrx-1 protein. Our result demonstrated that the content of free thiol groups specifically participating in disulfide bond formation in both hTrx-1-Trp31→Ala31 and hTrx-1-Trp31-deletion proteins was significantly lower than that in the wildtype hTrx-1 protein (Figure 4A). This suggested that the deletion/mutation of Trp31 in hTrx-1 inhibited the formation of disulfide bonds, thereby hindering the normal antioxidant function of hTrx-1.

Since the disulfide bond of the active site was always located at the end of the β2 chain on the molecular surface loop, we hypothesized that Trp31 may play a significant role in stabilizing the three β-chain turns near the active center during the formation of the stable Trx fold structure. Computational analysis using SPDBV software (version 4.10) revealed that in wild-type hTrx-1, there was a hydrogen bond between the C=O of Trp31 and the N-H of Gly33, and also between the C=O of Ala29 and the N-H of Cys32 (Figure 4B). We speculated that the presence of these two hydrogen bonds helps to stabilize the normal spatial structure of the active center. In hTrx-Trp31→Ala31, although the hydrogen bond between the C=O of Ala29 and the N-H of Cys32 still existed, the hydrogen bond between the C=O of Ala31 and the N-H of Gly33 disappeared (Figure 4C). In hTrx-1-Trp31-deletion, both of these hydrogen bonds were completely absent (Figure 4D). At the same time, in the reduced state of hTrx-1-Trp31-deletion protein, there was a slight change in the distance between the Cα atoms of Thr30 and Asp59/Met73 (Figure 4E–G). The positions of the β3 and β4 corner rings where Asp59 and Met73 are located are spatially close to the active center. Therefore, we speculated that these changes would affect the bimolecular nucleophilic substitution reaction of the antioxidant catalysis of hTrx-1, leading to a decreased capacity to form the disulfide bond between Cys32 and Cys35, eventually resulting in a significant decrease in the antioxidant activity of hTrx-Trp31→Ala31 and hTrx-1-Trp31-deletion proteins.

## 4. Discussion

Trx is a small-molecule protein existing in all biological cells, and its initial fundamental function lies in electron transport. The conversion of sulfhydryl/disulfide bonds of two cysteine (Cys) residues within its own molecule plays a pivotal role in maintaining intracellular redox homeostasis [23,24,25]. Over the lengthy process of biomolecular evolution, Trx has not only retained its most basic antioxidant activity but also progressively acquired numerous biological functions [26,27], including anti-apoptosis [28], regulation of transcription factors and gene expression, modulation of inflammatory responses, and cellular protection.

Leveraging conserved protein folds for the design of proteins tailored to perform specific functions represents a promising avenue of development in the era of artificial intelligence. Trx-1 serves as a paradigmatic instance, with its Trx fold structure being extensively utilized and investigated as a folding template [18]. The thioredoxin (Trx) superfamily, whose structure includes the Trx fold, is a large and diverse protein family. They function as protein disulfide oxidoreductases (PDOs) to regulate the redox state of target proteins through the reversible oxidation of dithiols in their active site. Members of the PDO family include Trx, glutaredoxin, protein disulfide isomerase (PDIs), TlpA, peroxiredoxin, NrdH redoxin, and bacterial DSB proteins families [29].

In these protein sequences, the most conserved sequence fragments are present in the active site and contain the conserved CXXC motif. The active-site disulfide bonds are always located at the end of the β2 chain on the molecular surface loop, followed by a long α-helix. Although the composition and properties of amino acid X in the CXXC motif vary greatly among different enzymes in the Trx subfamily, amino acid X is highly conserved in the Trx family. In addition, when introducing the conserved motif of the Trx protein active center in a large amount of the literature, only the CXXC motif is emphasized. However, in reality, whether in prokaryotic bacteria or in higher mammalian cells, its sequence is WCGPC (i.e., XCXXC).

The Trx antioxidant-catalyzed reaction is a bimolecular nucleophilic substitution reaction, which can be considered as a transfer of disulfide bonds from the substrate protein to Trx [30,31]. The N-terminal cysteine of the XCXXC motif is deprotonated and largely exposed at physiological pH, and is named the nucleophilic cysteine. Another cysteine is buried and typically protonated [32]. In different species of Trx, there are slight differences in the numbering of the positions where nucleophilic cysteine and buried cysteine are located. In mammalian and human Trx, nucleophilic cysteine and buried cysteine residues are located at positions 32 and 35, respectively [33].

Scientists have confirmed that Pro34 is the key residue determining the reducing ability of Trx-1 in this highly conserved motif, and has a significant impact on the stability of Trx-1 [34,35]. Is tryptophan, which is highly conserved at the N-terminus of the motif, also important? Considering that tryptophan is a hydrophobic amino acid that plays an important role in protein folding, membrane protein stability, and intermolecular interactions [36,37,38], and among the 105 amino acid residues of thioredoxin in mammals, including humans, although tryptophan only appears once (Trp31), its extreme conservation and active center position during evolution suggest that it plays an important role in the formation and function of thioredoxin’s active center structure. We therefore deleted the Trp31 site of human thioredoxin and replaced Trp31 with Ala, and obtained hTrx-Trp31→Ala31 and hTrx-1-Trp31-deletion proteins. Biochemical tests showed that compared to the wild-type Trx-1, the reducing ability of both mutant proteins decreased significantly. Multiple sequence alignment revealed that from lower prokaryotes to plants and higher mammals, besides the WCGPC motif of the antioxidant active center, there are more than 10 highly conserved amino acid residues (Figure 1A). Trx-1 structural analysis showed that these highly conserved amino acid residues are spatially clustered near the active center position after forming the Trx fold. Among them, three prolines have been subjected to mutation (substitution) analysis by scientists. The proline (Pro37 in *Staphylococcus aureus* Trx) on the α2 helix is not essential for the redox function of Trx, but is related to the stability of the protein structure [39]. The conserved cis-proline located opposite the active site motif (Pro73 in *Staphylococcus aureus* Trx) of WCGPC is crucial for maintaining the conformation of the active site and the redox potential of thioredoxin. Replacing it with alanine will affect catalytic efficiency [40]. The proline (Pro31 in *Staphylococcus aureus* Trx) located in the active site motif of WCGPC is a key residue that determines the reducing ability of thioredoxin. In *Escherichia coli* Trx, although replacing proline in the WCGPC motif with serine had only a slight effect on redox activity, replacing proline with histidine increased the redox potential relative to the wild type and significantly decreased antioxidant activity [40,41,42]. In *Staphylococcus aureus* thioredoxin, the substitution of proline in the WCGPC motif with serine or threonine leads to a decrease in the pKa of nucleophilic cysteine, an increase in redox potential, a decrease in catalytic efficiency, and a reduction in the stability difference between the oxidized and reduced forms [35].

We showed with hTrx-1 that replacing the tryptophan of the WCGPC active site motif by an alanine or deletion has a gigantic effect. It results in a decrease in the efficiency of catalysis, an extremely low ability to form disulfide bonds, and a decrease in stability of the mutant proteins compared to wild-type hTrx-1. Furthermore, we also provided evidence in eukaryotic cells. In vascular endothelial cells, oxidative stress elicits apoptosis, and our study demonstrated that the overexpression of Trx-1 mitigated this oxidative stress-induced apoptosis in the EA.hy926 cells. Notably, the deletion or mutation of the Trp31 in hTrx-1 diminishes its capacity to scavenge ROS and inhibit apoptosis.

Structural detail analysis had demonstrated that Trp31 resided on the corner ring of the β2 terminus of the active site, and its side chain’s indole ring acts like a “hat”, covering the corner ring at the end of β2 (Figure 1B). The aromatic benzene and pyrrole rings of the indole ring make tryptophan molecules significantly hydrophobic [36,37,43], providing a hydrophobic environment for the active center. Although alanine is a hydrophobic amino acid, its side chain methyl group is too small. It is speculated that the ability of alanine to provide a hydrophobic environment for the active center will significantly decrease after replacing Trp31. After Trp31 is deleted, the residue connected to the N-terminal cysteine is threonine, which is a hydrophilic amino acid. In addition, Asp60 and Met74 are located on the corner loops at the end of the β3 and β4 chains, respectively, and are very close in space to the corner loop at the end of the β2 chain where the active site is located. Multiple sequence alignment analysis further confirms that Asp60 and Met74 are highly conserved during the evolutionary process. We speculate that Trp31, Asp60 and Met74 may play important roles in stabilizing the three β-folding angle loops during the formation of Trx folding structures. However, in hTrx-1-Trp31-deletion protein, the distance between the Cα of the Thr30 center connected to the N-terminal cysteine and the Cα of the Asp59 and Met73 centers changed compared to the wild-type Trx (Figure 4E,G). Meanwhile, the hydrogen bonds that stabilize the spatial structure of the active center also decrease or disappear in hTrx-Trp31→Ala31 and hTrx-1-Trp31-deletion proteins in comparison to wild-type hTrx-1 (Figure 4B–D). These pieces of evidence indicate that after Trp31 is deleted or replaced by Ala, the hydrophobic environment of the active center is disrupted, and the spatial structural stability is affected. These changes may affect the bimolecular nucleophilic substitution reaction, resulting in a decrease in the ability to form disulfide bonds.

## 5. Conclusions

In this study, we demonstrated the significant role of the Trp31 residue in maintaining the correct conformation of the Trx active center, the antioxidant function of hTrx-1 and the redox defense ability of cells against oxidative stress. Mutating Trx-1 by replacing Trp31 with Ala31 (31A) or deleting Trp31 residues does not affect the effective expression of hTrx-1 protein in prokaryotic cells, but significantly reduces the antioxidant capacity of hTrx-1 and leads to a large number of cell deaths in EA.Hy926 human endothelial cells are associated with increased ROS production, and downregulation of antioxidant Nrf2 and HO-1 expression. In addition, we also found that the content of free thiol groups specifically participating in disulfide bond formation in Trp31 mutant hTrx-1 was significantly lower than that in wild-type hTrx-1, which may be due to the decreased ability of disulfide bond formation between Cys32 and Cys35. This study provides valuable insights into the importance of maintaining the antioxidant catalytic function of Trp31 residue in hTrx-1 during bimolecular nucleophilic substitution reactions.

## Data Availability

Data will be made available upon request.
